# Identification of novel growth phase- and media-dependent small non-coding RNAs in *Streptococcus pyogenes* M49 using intergenic tiling arrays

**DOI:** 10.1186/1471-2164-13-550

**Published:** 2012-10-13

**Authors:** Nadja Patenge, André Billion, Peter Raasch, Jana Normann, Aleksandra Wisniewska-Kucper, Julia Retey, Valesca Boisguérin, Thomas Hartsch, Torsten Hain, Bernd Kreikemeyer

**Affiliations:** 1 Institute of Medical Microbiology and Hospital Hygiene, University of Rostock, Schillingallee 70, 18057, Rostock, Germany; 2 Institute of Medical Microbiology, Genome Research, Justus-Liebig-University, Frankfurter Strasse 107, 35392, Giessen, Germany; 3 Systems Biology and Bioinformatics Group, University of Rostock, 18051, Rostock, Germany; 4 Genedata AG, Margarethenstrasse 38, 4053, Basel, Switzerland; 5 febit biomed GmbH, Im Neuenheimer Feld 519, 69120, Heidelberg, Germany

**Keywords:** *Streptococcus pyogenes*, Small noncoding RNAs, Virulence, Transcriptional regulation, Pathogenesis

## Abstract

**Background:**

Small non-coding RNAs (sRNAs) have attracted attention as a new class of gene regulators in both eukaryotes and bacteria. Genome-wide screening methods have been successfully applied in Gram-negative bacteria to identify sRNA regulators. Many sRNAs are well characterized, including their target mRNAs and mode of action. In comparison, little is known about sRNAs in Gram-positive pathogens. In this study, we identified novel sRNAs in the exclusively human pathogen *Streptococcus pyogenes* M49 (Group A *Streptococcus*, GAS M49), employing a whole genome intergenic tiling array approach. GAS is an important pathogen that causes diseases ranging from mild superficial infections of the skin and mucous membranes of the naso-pharynx, to severe toxic and invasive diseases.

**Results:**

We identified 55 putative sRNAs in GAS M49 that were expressed during growth. Of these, 42 were novel. Some of the newly-identified sRNAs belonged to one of the common non-coding RNA families described in the Rfam database. Comparison of the results of our screen with the outcome of two recently published bioinformatics tools showed a low level of overlap between putative sRNA genes. Previously, 40 potential sRNAs have been reported to be expressed in a GAS M1T1 serotype, as detected by a whole genome intergenic tiling array approach. Our screen detected 12 putative sRNA genes that were expressed in both strains. Twenty sRNA candidates appeared to be regulated in a medium-dependent fashion, while eight sRNA genes were regulated throughout growth in chemically defined medium. Expression of candidate genes was verified by reverse transcriptase-qPCR. For a subset of sRNAs, the transcriptional start was determined by 5^′^ rapid amplification of cDNA ends-PCR (RACE-PCR) analysis.

**Conclusions:**

In accord with the results of previous studies, we found little overlap between different screening methods, which underlines the fact that a comprehensive analysis of sRNAs expressed by a given organism requires the complementary use of different methods and the investigation of several environmental conditions. Despite a high conservation of sRNA genes within streptococci, the expression of sRNAs appears to be strain specific.

## Background

In recent years, the role of small non-coding RNAs (sRNAs) in regulation of bacterial gene expression has become more evident; however, the large number of sRNAs identified in different bacterial species was unexpected [1-3]. Even though sRNAs were conventionally regarded as inhibitory antisense regulators, a significant number of sRNAs that activate bacterial gene expression have been characterized [4]. Furthermore, regulatory mechanisms include both the stabilization and destabilization of target transcripts [5]. Bacterial sRNAs influence the expression of genes involved in processes as diverse as stress response, sugar metabolism, and surface composition [6-10]. With sRNAs representing a whole new level of post-transcriptional regulation, it is no surprise that these molecules play an important role in the tightly controlled expression of virulence factors in many pathogens [11,12].

We were interested in the regulatory sRNAome of *Streptococcus pyogenes* (group A streptococci, GAS), a common, exclusively human pathogen that causes a variety of diseases. GAS is responsible for mild superficial infections of the skin (impetigo contagiosa) and mucosal membranes (pharyngitis and tonsillitis). Additionally, there is a high global burden of severe GAS diseases such as post-streptococcal sequelae, and severe systemic (streptococcal toxic-shock-like syndrome) or invasive infections (necrotizing fasciitis), leading to over 500,000 deaths per year [13,14]. The controlled expression of virulence factors plays a role in GAS infection, persistence in the host, and development of invasive diseases, which makes the investigation of virulence factors and their regulation a research priority. GAS expresses a large number of virulence factor genes coding for a variety of proteins, including surface components, lytic enzymes, proteinases, cytotoxins, superantigens, and immunoprotective proteins, that are controlled at least partially by the 30 stand-alone transcriptional regulators and 13 two-component systems identified to date in GAS [15]. Virulence factor expression in GAS is highly responsive to environmental conditions and greatly depends on the growth phase.

Little is known, however, about the importance of sRNAs for virulence-related gene regulation in GAS. An overview of small RNAs in streptococci is nicely presented by Le Rhun and Charpentier [16]. Several individual sRNAs have been identified in GAS [17,18], with many more predicted by bioinformatic screens [12,19]. Previous analysis of sRNA expression in a GAS M1T1 serotype using an intergenic tiling array approach identified 40 potential sRNAs, with a very low predicted overlap with candidate genes [20]. The authors concluded that sRNA expression in GAS is serotype-dependent. The current work focused on sRNA expression in the skin isolate GAS M49. An intergenic tiling array identified 42 novel and 13 known sRNAs. Data from this experiment were compared to the results of the former GAS M1T1 study, and to predictions of two recently published bioinformatics tools [19,21]. Additionally, we tested the regulation of sRNA expression in correlation to growth media and growth phase. We found very little overlap between the different screening methods, which underlines the importance of using several complementary methods, as well as several environmental conditions, to attain a comprehensive analysis of bacterial sRNAomes.

## Results

### Identification of sRNAs in the intergenic regions of GAS M49

Custom intergenic tiling arrays representing the genome of *S. pyogenes* NZ131 (NCBI accession number: NC_011375) were designed to detect the expression of potential sRNAs. A total of 17,823 50-mer probes with an overlap of 15 bp were synthesized to cover the intergenic regions, with 9,082 probes representing the positive strand and 8,741 probes covering the negative strand. Additionally, 174 probes were designed as control probes covering tRNA genes or genes coding for known sRNAs. For example, we probed for *fas*X [17], SR914400, and SR1754950 [20], all of which were detected in our experiments. Genedata Selector software was used to integrate genomes, tiling array probe sequences, sRNA predictions, and experimental data. Expression data were analysed in their genomic- and sequence-based contexts (Genedata AG).

Total RNA for the tiling array experiments was isolated from GAS M49 grown in chemically defined medium (CDM). Samples were taken from four biological replicates during both mid-log growth phase (OD_600_= 0.4–0.6) and stationary growth phase (OD_600_= 1.2). A signal intensity of >300 was set as a threshold. A positive signal required that a minimum of one probe specific for one strand showed an intensity above the threshold in at least three replicates. Intergenic regions featuring high intensities on both strands were manually removed following the analysis.

**Figure 1:**
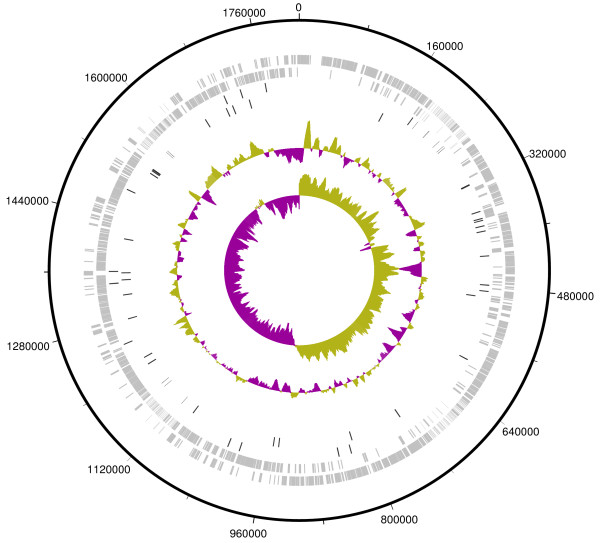
**Circular representation of the GAS M49 genome.** Tracks from outermost moving inward: Track 1, NZ131 genome; Tracks 2 and 3, presence of coding genes on the forward and reverse strand, respectively; Tracks 4 and 5, sRNA genes forward and reverse, respectively. Tracks 6 and 7 show the GC plot and the GC skew of the genome.

We identified a total of 55 putative sRNAs in GAS M49 that were expressed during growth in CDM, 42 of which were novel. Computational functional prediction revealed that a subset of the newly identified RNAs included molecules with similarities to one of the common non-coding RNA families included in the Rfam database [22]. The database covers functional categories of non-coding RNAs determined from multiple sequence alignments. Using the Rfam database, we predicted functions for 14 putative sRNAs. One of these RNAs was predicted to be the structural RNA of the bacterial signal recognition particle (SRP), and another was predicted to be the bacterial RNase P RNA. Further functional categories included three T-box leader elements, three CRISPR family members, one tmRNA, and one endoribonuclease, RNaseP_bact_b (Table 1). Table 1 contains a summary of the information pertaining to all 55 candidate sRNAs, including the flanking genes, Rfam prediction, and conservation across other genomes. Five putative sRNA sequences overlapped with adjacent ORFs on the same strand. Functional studies will be necessary to clarify whether the corresponding sRNAs are transcribed independently. The overall GC content of all 55 sRNA candidate sequences was 38.3%. This correlates with the GC content of the whole NZ131 genome (38.0%). No specific strand prevalence and no clustering in specific genomic regions were observed for the regulatory sRNA genes. The replication-related gene orientation bias of the protein coding genes [23,24] was mirrored by the sRNA gene candidates. A circular depiction was created with the Artemis DNAPlotter tool [25] (Figure 1) to visualize the sRNA genes in the context of the NZ131 genome.

**Table 1:** ***S. pyogenes *M49 sRNAs, genomic location, Rfam predictions, and conservation across streptococcal genomes**

**No**	**ID**	**Left nucleotide**	**Right nucleotide**	**Size (nt)**	**Strand**	**Upstream adjacent gene**	**Downstream adjacent gene**	**Rfam and MOSES prediction**	**Previously published**	**Conservation**
1	sRNASpy490131	141429	141478	49	-	ntpK	ntpE			a
2	sRNASpy490186c	194707	194791	84	+	Spy49_0186c	speG	SRP_bact	[37]	c
3	sRNASpy490206	216807^d^	217021	215	+^e^	fasA	rnpA	MOSES2	fasX [17]	b
4	sRNASpy490229	238850	238899	49	+	prgA	rpsL			b
5	sRNASpy490238	248530	248579	49	+	Spy49_0238	bacA			a
6	sRNASpy490241	251595	251644	49	+	rgpG	Spy49_0242			a
7	sRNASpy490305	318826	318910	84	+	Spy49_0305	Spy49_0306	23S-methyl		b
8	sRNASpy490306	319622	319776	154	+	Spy49_0306	Spy49_0307	FMN	RNA319780 [20]	b
9	sRNASpy490348	362857	362906	49	+	Spy49_0348	Spy49_0349c	MOSES6		a
10	sRNASpy490366c	373806	373855	49	+	Spy49_0366c	Spy49_0367c			-
11	sRNASpy490370	376843	376892	49	+	Spy49_0370	Spy49_0371			a
12	sRNASpy490380c	385431 ^d^	385526	95	+ ^e^	Spy49_0380c	mtsA			b
13	sRNASpy490388	393522	393571	49	+	rplA	pyrH			a
14	sRNASpy490434	431752	431836	84	+	Spy49_0434	thrS	T-box		b
15	sRNASpy490483c	476503^d^	476600	97	+ ^e^	Spy49_0483c	bglG			b
16	sRNASpy490493	486133	486182	49	+	Spy49_0493	ptsK			a
17	sRNASpy490504	495351	495400	49	-	Spy49_0504	Spy49_0505c			a
18	sRNASpy490592	594852	594936	84	+	Spy49_0592	pheS			b
19	sRNASpy490727	733841	733890	49	-	Spy49_0727	Spy49_0728			a
20	sRNASpy490822	820820	820996 ^d^	176	- ^e^	Spy49_0822	Spy49_0823	CRISPR	RNA772970 [20]	c
21	sRNASpy490827	827456	827785	329	+	Spy49_0827	Spy49_0828	CRISPR		
								MOSES14		a
22	sRNASpy490845c	846977	847061	84	+	trmE	rplJ	L10_leader		c
23	sRNASpy490948c	941583	941657 ^d^	74	-	Spy49_0948c	guaA			a
24	sRNASpy490957c	950434	950518	84	- ^e^	Spy49_0957c	Spy49_0958c		SR914400 [20]	b
25	sRNASpy491007c	1000709	1000828	119	+	pcrA	Spy49_1008	Glycine		b
26	sRNASpy491023c	1019382	1019746	364	+	Spy49_1023c	Spy49_1024	tmRNA	RNA983400 [20]	b
27	sRNASpy491061c	184794	184878	84	+	Spy49_1061c	Spy49_0176			c
28	sRNASpy491095c	1092894	1092943	49	-	ptsH	nrdH			c
29	sRNASpy491122c	1117169	1117218	49	-	sodM	holA			b
30	sRNASpy491163c	1156488	1156537	49	-	Spy49_1163c	clpE			a
31	sRNASpy491167c	1163270	1163319	49	-	ileS	divIVA	T-box		-
32	sRNASpy491206c	1201615	1201734	119	-	ccdA	Spy49_1207c	MOSES16	RNA1239700 [20]	a
33	sRNASpy491217c	1214010	1214059	49	-	Spy49_1217c	Spy49_1218c		SR1251900 [20]	b
34	sRNASpy491243	1246370	1246419	49	+	Spy49_1243	Spy49_1244			a
35	sRNASpy491275c	1282084	1282413	329	-	Spy49_1275c	Spy49_1276c	RNaseP_bact b	RNA1320100 [20]	c
36	sRNASpy491311c	1321638	1321853^d^	84	- ^e^	glyQ	Spy49_1312c			b
37	sRNASpy491336c	1341554	1341603	49	+	rbfA	infB			b
38	sRNASpy491340c	1346300	1346349	49	-	nusA	Spy49_1341c			b
39	sRNASpy491354	1357803	1357852	49	-	manL	manM			b
40	sRNASpy491357	1360204	1360323	119	+	Spy49_1357	serS	T-box	RNA1439100 [20]	b
41	sRNASpy491409c	1413359	1413408	49	-	acpS	secA			a
42	sRNASpy491489c	1489011	1489060	49	+	Spy49_1489c	Spy49_1491c			b
43	sRNASpy491555c	1528842	1528891	49	-	Spy49_1555c	Spy49_1556c			a
44	sRNASpy491560c	1533843	1533892	49	-	Spy49_1560c	pyrG			a
45	sRNASpy491561c	1535755	1535804	49	-	pyrG	rpoE			c
46	sRNASpy491562c	1536613	1536662	49	-	rpoE	tig			a
47	sRNASpy491591	1571896	1571945	49	-	lacR2	Spy49_1592	MOSES18	SR1604140 [20]	a
48	sRNASpy491596c	1574723	1574807	84	-	rplM 50S	Spy49_1597c	L13_leader		c
49	sRNASpy491671c	1654859	1654908	49	-	emm49	Spy49_1672c			a
50	sRNASpy491707c	1693440	1693489	49	-	Spy49_1707c	Spy49_1708			a
51	sRNASpy491713	1698142	1698191	49	+	Spy49_1713	Spy49_1714c		SR1719800 [20]	a
52	sRNASpy491718c	1704471	1704525	54	-	ctsR	csp			a
53	sRNASpy491732c	1724321	1724370	49	+	Spy49_1732c	rpsB		SR1745900 [20]	b
54	sRNASpy491738	1733300	1733433^d^	133	-	Spy49_1738	Spy49_1739c		SR1754950 [20]	b
55	sRNASpy491769c	1763496	1763545	49	+	hisS	rpmF			b

Coordinates and gene designations of adjacent genes are relative to the *S. pyogenes* NZ131 genome (NCBI accession number: NC_011375).

^a^*Streptococcus pyogenes.*

^b^ Beta-haemolytic streptococci.

^c^ Non-beta-haemolytic streptococci.

^d^ Transcriptional start site determined by 5^′^ RACE.

^e^ Strand confirmed by gene specific RT-PCR.

In a previous bioinformatic approach (MOSES), 20 probable candidates were predicted [19]. In our array analysis, expression of five of the predicted sRNAs was confirmed under the conditions studied (Table 1). Furthermore, we detected 12 streptococcal RNAs that were previously identified by Perez et al. [20] (Table 1). The phylogenetic conservation of the putative sRNAs was tested by BLAST analysis, and the taxonomic classification was presented following the nomenclature of Facklam [26]. The tiling array technique with overlapping probes did not allow us to detect an accurate start site for the respective sRNA genes. Thus, we described the nucleotides represented by the active probes as the preliminary start and end (Table 1).

**Figure 2:**
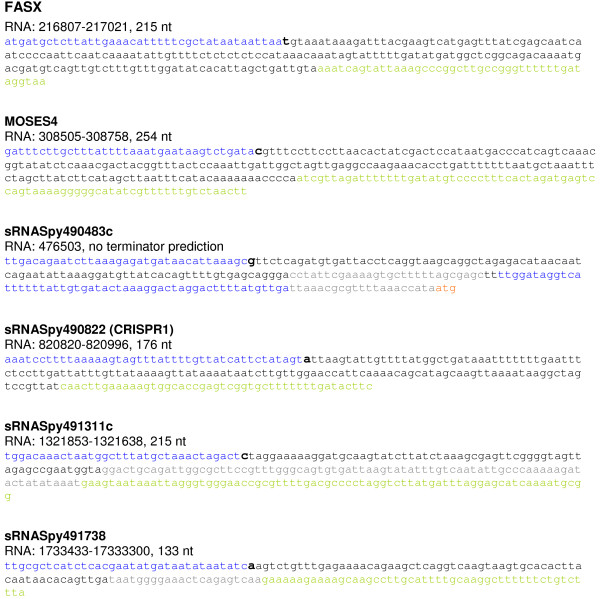
**Determination of the transcriptional start site of putative sRNAs.** Transcriptional start sites were detected by 5^′^ RACE-PCR and are depicted in black/bold. The sequenced sRNA sequence is depicted in black, predicted RNA sequences are depicted in grey, predicted promoter regions are depicted in blue, predicted rho-independent transcription terminators are depicted in green. The start codon of down-stream genes is depicted in orange.

For a subset of six candidate sRNA genes, we determined the transcriptional start site (TSS) of the sRNA molecules using the 5^′^ rapid amplification of cDNA ends- (5^′^ RACE) technology (Invitrogen). The TSS of the analysed sRNAs is shown in Table 1. 5^′^ RACE was conducted for two known sRNA genes, *fasX* and sRNASpy490822 (CRISPR1), for one candidate predicted by MOSES (MOSES4), and for three novel sRNA candidates, sRNASpy490483c, sRNASpy491311c, and sRNASpy491738. The results of the 5^′^ RACE analysis are shown in Figure 2. Promoter and terminator predictions for the respective sRNA candidate genes are also included.

### Comparison of the sRNA expression data using different sRNA screens

**Figure 3:**
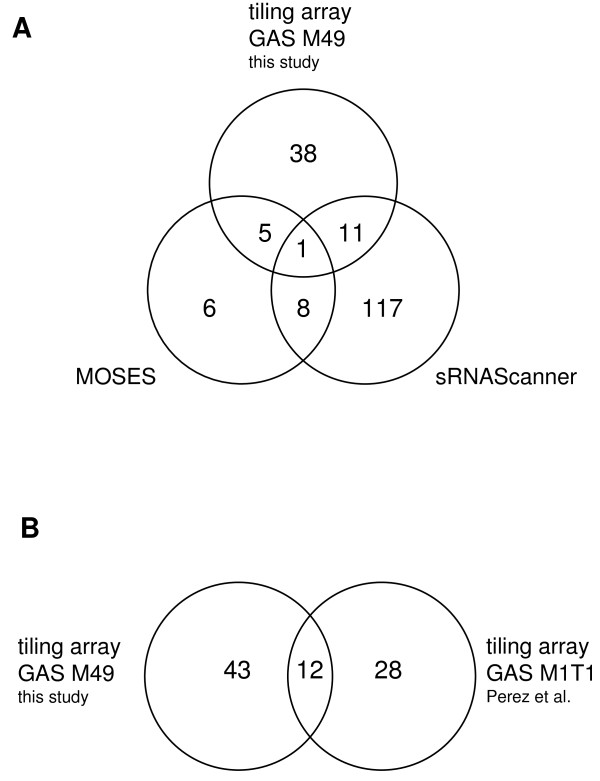
**Venn diagrams describing the relationships between three different sRNA screens of two *S. pyogenes *genomes.A**: Comparison of the outcome of two bioinformatics screens and one tiling array analysis of the *S. pyogenes* NZ131 genome. The total number of candidates is shown for each of the three screens. Candidates identified in more than one screen are represented by the respective overlapping sections. **B**: The total number of candidates is shown for two tiling array experiments in *S. pyogenes* M49 (this study) and M1T1 [20], respectively. Candidates identified in both screens are shown in the overlapping section.

The tiling array data were compared with the prediction results of two recently published bioinformatics tools, sRNAScanner [21] and MOSES [19]. As shown in Figure 3A, the overlap between the GAS M49 array data and the sRNA predictions performed with the sequence of the NZ131 genome was minimal. From 20 MOSES candidates, five were detected in the tiling array analysis, while from 137 sRNAScanner predictions, 11 showed a signal in the array. Eight putative sRNAs were selected by both programs. There was only one sRNA that was predicted by both algorithms and was also detected in the tiling array (Figure 3A). The general accuracy of the three independent screens was supported by the fact that this mutual candidate was the known streptococcal sRNA FASX [17].

We also compared our tiling array data with a previously published sRNA microarray study in GAS M1T1 [20]. The previous study identified 40 putative sRNAs. Even though the sequences of the candidates were conserved across streptococcal genomes, serotype-specific variation of sRNA transcript abundance was observed in northern blot experiments. Screening of GAS M1T1 was conducted with cells grown in complex medium [20], whereas the expression experiments in this study were performed with GAS M49 grown in CDM. Consequently, we found only a limited overlap between the two microarray screens (Figure 3B). Twelve sRNA candidates were detected in both strains.

### Analysis of common motifs in the GAS M49 sRNA population

**Figure 4:**
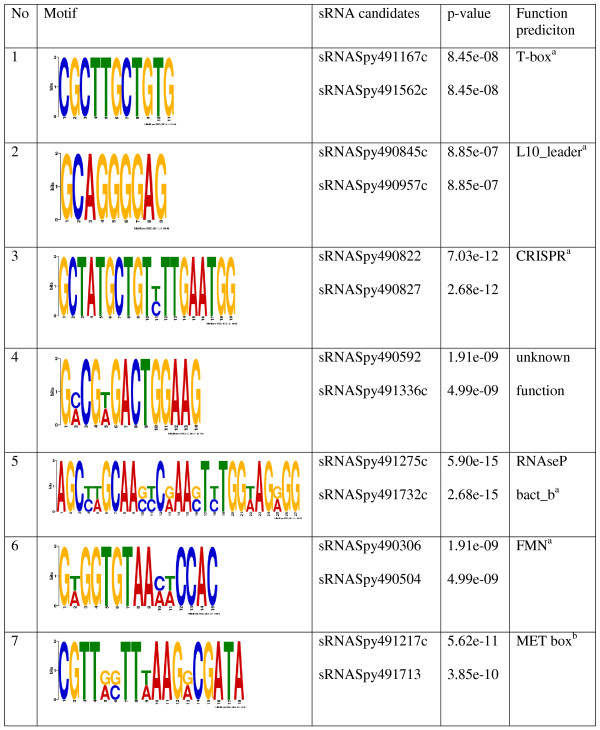
**Shared sequence motifs in sRNA candidates.**^a^Rfam, ^b^TOMTOM.

To identify putative functional regions within the sRNA candidates, all sequences were screened for common features by motif-based sequence analysis using MEME SUITE [27]. The occurrence of shared sequence motifs in different sRNA species could be an indication of common structural properties with functional significance in this region. Seven motifs were identified with consensus sequences with *p*-values < 1.0 ×10^-7^, spanning 9–27 base pairs (Figure 4). Putative motif sequences were compared to members of the Rfam database families, and were subjected to TOMTOM [28] motif analysis using the RegTransBase prokaryotic database. Candidates sRNASpy490592 and sRNASpy491336c shared a 14 bp consensus sequence with no apparent known function (motif 4, Figure 4). For all other identified motifs, a known function could be assigned to the respective candidates. The corresponding RNAs were either predicted to be RNAs with non-regulatory functions, e.g. RNAseP (motif 5, Figure 4), or more typically, to represent *cis*-regulatory RNA elements, e.g. FMN riboswitch or MET box (motif 6 and motif 7, Figure 4).

### Regulated expression of sRNA genes in GAS M49

**Figure 5:**
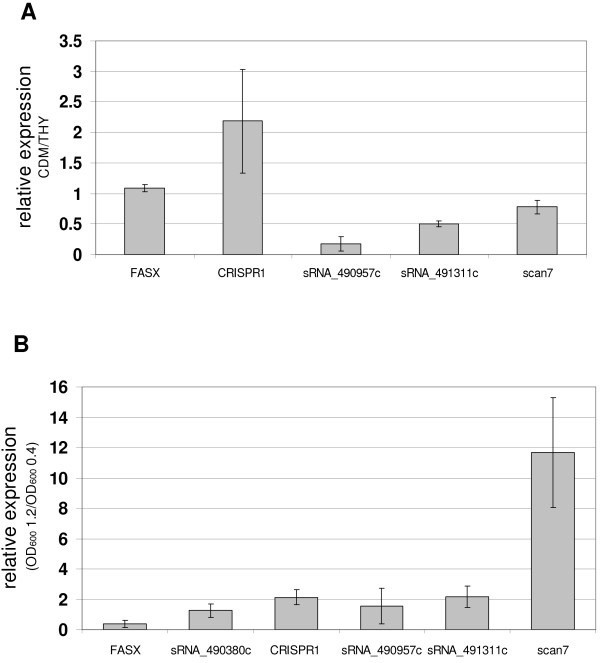
**Expression of sRNA candidates verified by qRT-PCR analysis.A**: sRNA expression during growth in CDM compared to growth in THY. Samples were taken during the exponential growth phase (OD_600_ = 0.4). Relative sRNA gene expression in CDM is depicted in this graph (expression in THY is normalised to one). **B**: sRNA gene expression throughout growth in CDM. Samples were taken in the exponential growth phase (OD_600_ = 0.4) and in the stationary phase (OD_600_ = 1.2). Relative sRNA gene expression at OD_600_ = 1.2 is depicted in this graph (OD_600_ = 0.4 is normalised to one).

Regulation of sRNA gene expression in GAS M49 under different growth conditions was studied by intergenic tiling array analyses. Total RNA was isolated from bacteria grown in CDM, BHI, or THY. Samples were collected in the exponential and stationary phases. Transcript level changes were expressed as the log_2_ signal ratio between conditions, and are listed in Table 2. sRNA gene expression was considered significantly different when the log_2_ ratio of the signals was ≤ −1.58 or ≥ 1.58. Twenty-four sRNA genes were regulated in a growth phase- and/or medium-dependent fashion. During growth in CDM, five genes were up-regulated in the stationary growth phase compared to the exponential growth phase, whereas three genes were down-regulated. One of the down-regulated sRNA genes was *fasX*. This is in accord with previous results, where a reduction in *fasX* transcript abundance in the stationary growth phase was detected by northern blot analysis [17]. This observation was also confirmed by qRT-PCR analyses (Figure 5B). Comparison of sRNA gene expression during growth in THY with expression during growth in CDM revealed differential expression of 17 sRNA genes. Of these, 13 genes were down-regulated and four up-regulated in THY. Growth in BHI led to the detection of 12 media-dependent controlled sRNA genes. Nine genes were down-regulated and three were up-regulated during growth in BHI compared to growth in CDM. From the 20 sRNA genes that showed media-dependent regulation, seven were regulated in both THY and BHI, and showed the same direction of regulation compared to CDM. Twelve sRNA genes were exclusively regulated in one of the two media, and only one gene was down-regulated in THY but up-regulated in BHI compared to CDM. These results are in accord with the fact that both media THY and BHI are complex media, as opposed to the synthetic medium CDM, which forces the bacteria to synthesize a number of components essential for growth. Thus, in CDM, changes in bacterial metabolism are necessary for successful growth and require adaption of the bacterial transcriptome, including the sRNAome.

**Table 2:** **Regulation of *S. pyogenes *M49 sRNA gene expression dependent on culture medium and growth phase**

**No**	**ID**	**THY vs CDM exponential growth phase**	**THY vs CDM stationary growth phase**	**BHI vs CDM exponential growth phase**	**BHI vs CDM stationary growth phase**	**Stationary vs exponential in CDM**
1	sRNASpy490186c		−2,72			
2	sRNASpy490206	−1,78				−2,00
3	sRNASpy490238	3,33				
4	sRNASpy490305	−2,50	−3,03	−2,10	−3,59	
5	sRNASpy490306				1,72	
6	sRNASpy490348	3,42				
7	sRNASpy490370				−2,51	
8	sRNASpy490380c		−3,02	−2,28	−3,82	
9	sRNASpy490493	2,18				
10	sRNASpy490592					1,81
11	sRNASpy490827	−1,89				
12	sRNASpy490845c				−1,98	2,03
13	sRNASpy490957c		−2,67			
14	sRNASpy491007c		−1,73		−1,81	1,94
15	sRNASpy491023c	−1,84		1,92	3,54	
16	sRNASpy491206c	1,58			2,65	−2,41
17	sRNASpy491311c					−2,20
18	sRNASpy491561c	−1,99				
19	sRNASpy491596c		−1,60	−1,88		
20	sRNASpy491707c					1,68
21	sRNASpy491713					1,76
22	sRNASpy491718c			−1,71		
23	sRNASpy491732c		−2,38		−3,60	
24	sRNASpy491738		−3,40	−2,07	−4,30	

### Validation of sRNA expression by qRT-PCR and northern blot analyses

Expression of sRNA candidates by GAS M49 was tested by gene-specific reverse transcription followed by real-time PCR analysis. Experimental expression validation was performed for the RNAs FASX and sRNASpy490822 (CRISPR1), for the sRNA scan7, which was predicted using sRNAscanner [21], and for three more candidates identified by tiling arrays in this study (sRNASpy490380c, sRNASpy490957c, and sRNASpy491311c). The expression of the candidates in GAS M49 was verified. Moreover, we confirmed the orientation of the sRNA genes by employing single gene-specific primers for the reverse transcription reaction. Three reactions were performed in parallel: one including the forward primer, one including the reverse primer, and one without any primers. Signals were only detected in samples containing the primer complementary to the coding strand of the respective gene (data not shown). We compared sRNA expression of GAS M49 cultured in CDM medium and THY broth throughout growth (Figure 5). 5S RNA was used as an internal control for normalization, and was expressed in comparable amounts under all conditions tested in this experiment. Expression of *fasX* was equivalent during growth in CDM and THY (Figure 5A). FASX was down-regulated in the stationary phase, an observation that confirmed the array data and previously published results from northern blot analyses [17]. We did not detect strong regulation of CRISPR1, sRNASpy490957c, or sRNASpy491311c during growth in CDM (Figure 5B). In contrast, scan7 was highly up-regulated in the stationary phase (Figure 5B). The expression of sRNASpy490380c was much higher in THY compared to CDM (almost 100-fold, data not shown). During growth in CDM, no changes in the low level expression of sRNASpy490380c were observed (data not shown).

**Figure 6:**
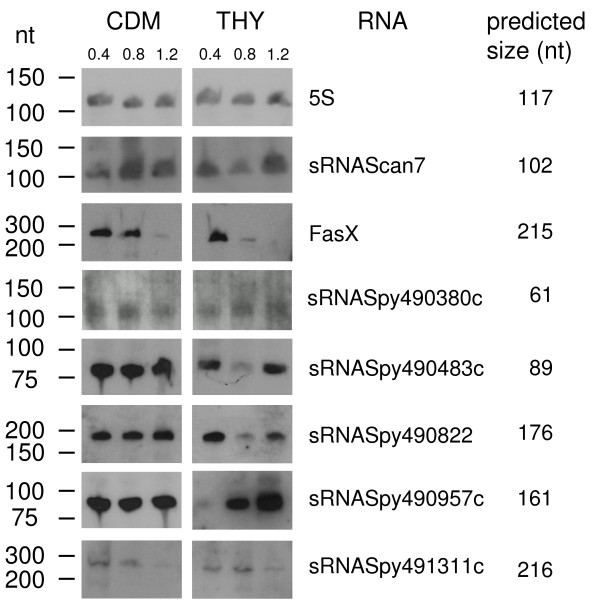
**Confirmation of sRNA candidate expression by northern blot analysis.** sRNA expression during growth in CDM and in THY medium. 0.4: exponential growth phase (OD_600_ = 0.4); 0.8: transitional growth phase (OD_600_ = 0.8); 1.2: stationary growth phase (OD_600_ = 1.2). The probes were specific for the RNAs indicated on the right of each blot. For comparison, the approximate sizes of the sRNA candidates, as determined by 5′RACE analysis, are indicated on the far right.

To further verify candidate gene expression, northern blot analysis of the same putative sRNA genes was performed (Figure 6). This method allows the determination of approximate transcript sizes. Probes specific for 5S RNA and FASX were included as controls. The apparent molecular weight of candidates CRISPR1, sRNASpy490380c, sRNASpy490483c, sRNASpy491311c, and scan7 corresponded to the length predicted by 5^′^ RACE determination. The CRISPR transcript, tracrRNA, showed a band at the expected size of 176 nucleotides, as well as several smaller bands that were likely the result of RNA processing, as observed previously in GAS M1T1 [29] ( Additional file 1A). For the putative sRNASpy490957c, transcript analysis by 5^′^ RACE predicted a 161 nt full-length product, including the terminator region. However, the most prominent band detected by northern blot analysis migrated at approximately 80 nt. Low intensity bands were detected at approximately 90 nt and 160 nt ( Additional file 1B), which might indicate post-transcriptional sRNA processing.

## Discussion

Bacterial gene regulation by sRNAs has gained a lot of attention in recent years, because it plays an important role in many cellular processes, including response to environmental changes, growth, and pathogenesis. There is an intriguingly large diversity of regulatory mechanisms, including *cis*- and *trans*-acting sRNAs, untranslated regions, and riboswitches. Some sRNA molecules act as repressors of translation and destabilize mRNA transcripts, but others act by activating and stabilizing target mRNAs [30-32]. One of the best characterized sRNAs in GAS is FASX, which is involved in virulence-related gene regulation [17,33]. Knock-out mutants of *fasX* show a reduced expression of secreted virulence factors such as streptokinase and streptolysin S. The mechanism for streptokinase gene (*ska*) expression control is the stabilization of the *ska* transcript [33]. Lack of FASX-*ska*-mRNA-interaction in the *fasX* deletion mutant decreased transcript levels, and consequently decreased streptokinase protein abundance.

A second example of a regulatory RNA in GAS is the untranslated mRNA of the streptococcal pleiotropic effect locus (*pel*), which contains *sagA*, the structural gene for streptolysin S. This region was described as a positive regulator of important streptococcal virulence factors, including M-protein, Sic, and SpeB [34]. Strain specificity of PEL function is indicated by the fact that *emm* transcription was not affected in a *sagA*-deficient mutant with a M6 background [35]. Similar results have been obtained in GAS M1 and M18 Tn916 *sagA* mutant strains [36]. Additionally, *pel* deletion mutant analysis of four M1T1 GAS isolates did not identify any regulatory function for the *pel* sRNA in this serotype [20].

Another, more recently described untranslated RNA with influence on streptococcal virulence is the 4.5S RNA, a component of the bacterial signal recognition particle (SRP) [37]. While the 4.5S RNA gene is not essential, mutation impairs bacterial growth, lowers virulence factor secretion, and reduces virulence in a mouse infection model.

Recently, several whole genome sRNA screens in Gram-positive bacteria, employing either tiling array or next generation sequencing approaches, revealed an unexpected number of potential sRNAs in several pathogenic species [38-42]. In this context, it is likely that GAS expresses more sRNAs responsible for virulence gene expression control. One whole-genome intergenic tiling array screen of GAS M1T1 identified approximately 40 sRNAs that were expressed during the exponential growth phase in cells cultivated in THY complex medium [20]. The GAS M49 sRNAome in the present study was determined using cells grown in CDM. From 55 putative sRNAs in GAS M49, only 12 were detected previously in the GAS M1T1 screen (Figure 3B). This result is in accord with the concept that sRNA expression is serotype-dependent and regulated by environmental stimuli. Consequently, we detected media- and growth-phase-dependent sRNA gene regulation in the tiling array expression analysis, or by qRT-PCR of selected candidate genes. It would be interesting to monitor sRNA gene expression regulation under infection-relevant conditions.

Clustered, regularly interspaced, short palindromic repeat (CRISPR) loci represent an adaptive RNA-based immune system that protects bacteria and archaea from horizontal transfer of phage and plasmid DNA [43]. Among the putative sRNA genes detected in GAS M49 by the tiling array approach, two sequences were categorised by the Rfam prediction program as CRISPR-related RNAs (Table 1). sRNASpy490822 and sRNASpy490827 are encoded by the system II (Nmeni/CASS4 subtype) [44] CRISPR/Cas locus, which was characterized recently by differential RNA sequencing in GAS SF370 (M1 serotype) [29]. Our data suggest that this locus is also active in GAS M49. Expression of sRNASpy490822 was confirmed by RT-PCR on the opposite strand of the CRISPR-associated genes under all conditions tested in this study. This transcript corresponds to the *trans*-activating CRISPR RNA (tracrRNA), which is responsible for the maturation of CRISPR RNA in concert with RNase III and the CRISPR-associated Csn1 protein [29]. A third CRISPR-related RNA detected in our expression screen, sRNASpy491206c, is encoded in the system I-C (Dvulg/CASS subtype) [44] CRISPR/Cas locus, which is also conserved in streptococcal genomes. In contrast to our array data, this locus appeared to be silent in GAS SF370, where no expression was detected in the differential RNA sequencing approach [29]. Even though the CRISPR loci are conserved throughout GAS genomes, the activity of different CRISPR subtypes appears to be serotype-specific.

In the early years of sRNA research, many bioinformatic prediction tools were developed. One of the most prominent programs was the SIPHT tool, which has been used for many bacterial species [45,46]. However, comparison of the prediction results with the actual *in vivo* expression of sRNAs often revealed very little overlap between the different screening methods [20,41,47]. The reasons for this discrepancy may be the limitations of the prediction programs as well as the fact that not all sRNAs are expressed under all conditions. The development of sRNA prediction software with improved properties is on-going. We compared our tiling array data with the prediction results of two recently published bioinformatics tools, sRNAScanner [21] and MOSES [19]. As depicted in Figure 3A, the overlap between the tiling array expression data and the sRNA predictions was low. From the 20 most probable candidates of the MOSES analysis, 25% were expressed in GAS M49, whereas 8% of the predicted sRNAScanner predictions were found in the array analysis. Even the overlap between the two bioinformatics data sets was low. The only sRNA that was detected in all three screens was the previously characterized sRNA FASX [17]. These results strongly suggest that a comprehensive analysis of bacterial genomes requires the combination of mathematical predictions with the collection of expression data. In the long term, testing of different conditions, especially mimicking *in vivo* situations by employing infection models, might lead to an increased overlap of expression detection and bioinformatics analyses.

## Conclusions

We present here the identification of 55 putative sRNAs in GAS M49 by an intergenic tiling array approach. The candidate sRNA genes were expressed during growth in CDM. Forty-two of the RNAs were novel, whereas 13 RNAs have been described previously. The sequences of most of the candidates were conserved over streptococcal genomes. However, comparison of our GAS M49 sRNA expression data to another array analysis of a GAS M1 strain, and to two *in silico* screening methods, revealed little overlap between the different approaches. Thus, the investigation of several conditions and the combination of screening tools will be necessary to gain a comprehensive understanding of the abundance of sRNAs in GAS. The identification of novel differentially expressed sRNA genes will enhance our understanding of virulence related gene regulation in GAS. To account for specific expression patterns of putative sRNAs, infection relevant conditions combined with next generation RNA sequencing should be employed to investigate sRNA dependent regulatory networks in GAS.

## Methods

### Bacterial strains and culture conditions

GAS serotype M49 strain 591, a clinical isolate from a skin infection, was kindly provided by R. Lütticken (Aachen, Germany). The GAS strain was cultured in chemically defined medium (CDM) [48], Todd-Hewitt broth (Invitrogen) supplemented with 0.5% yeast extract (THY; Invitrogen), or Brain-Heart-Infusion medium (BHI; Oxoid), as indicated, at 37°C under a 5% CO_2_/20% O_2_ atmosphere.

### RNA isolation

Total bacterial RNA from cultures grown to exponential and stationary phase of growth was isolated using the FastRNAProBlue Kit from MP Biomedicals according to manufacturer’s instructions. The purified total RNA was digested with DNaseI (Ambion) to remove remaining traces of chromosomal DNA. The RNA preparation was treated with 10 U of DNase1 for 30 min at 37°C. The enzyme was subsequently heat inactivated at 72°C for 5 min.

### Enrichment of small RNAs

Five micrograms of total RNA were fractionated using the Ambion FlashPAGE Fractionator, Ambion FlashPAGE Precast Gels, and the Ambion FlashPAGE Buffer Kit, following manufacturer’s instructions. To collect the fraction of RNA molecules <200 nucleotides in length, the protocol was modified by increasing the running time from 12 min to up to 45 min at 75 V.

### Labelling

The small RNA fraction was ethanol-precipitated overnight at −20°C. The RNA was pelleted by centrifugation, dissolved in nuclease-free water, and labelled using the Ambion mirVana miRNA Labeling Kit following the manufacturer’s instructions. In brief, this kit involves two main steps; the 3^′^ amine-modified tailing reaction, and labelling with NHS-esters. Poly(A) Polymerase and a mixture of unmodified and amine-modified nucleotides were used to add a 20–50 nucleotide tail to the 3^′^ end of each RNA molecule in the sample. The amine-modified RNA molecules were purified and coupled to amine-reactive labelled biotin moieties as NHS-esters.

### Design and synthesis of microfluidic microarrays

We used a microfluidic biochip (Febit Biomed) consisting of eight independent reaction chambers, the arrays, enclosed in a cartridge for fully automated processing. Each array contains 15,625 features which are synthesized *in situ* inside the microchannels using the Geniom One technology (febit biomed) [49]. The 50mer probes were designed as a whole genome tiling array, covering the intergenic regions of the *S. pyogenes* NZ131 genome (NCBI accession number: NC_011375). The forward and reverse oriented probes were synthesized in separate arrays. Thus, two arrays per sample were used.

### Microarray hybridization and detection

All hybridization and detection steps were carried out using a Geniom RT Analyzer (febit biomed). Hybridizations were performed overnight (16 hours) at 42°C. Subsequently, biotin was detected with streptavidin-phycoerythrin (SAPE). A signal amplification step was added using biotinylated anti-streptavidin antibodies (Vector Laboratories) and a second incubation with SAPE (Invitrogen). Signal detection using the appropriate filter set (Cy3) of the Geniom device employed the auto-exposure function of the Geniom software. The data discussed in this publication have been deposited in the NCBI Gene Expression Omnibus [50] and are accessible through GEO Series accession number GSE31228 ( http://www.ncbi.nlm.nih.gov/geo/query/acc.cgi?acc=GSE31228).

### Microarray data analysis

Raw intensities were analysed and extracted using Geniom Wizard software (febit biomed) as a tab delimited text file. The data were then converted into a matrix, with rows corresponding to the features and columns corresponding to the different samples. Data analysis was performed using GeneSpring GX (version 11) software (Agilent Technologies). The array background was calculated as the median signal intensity of all “blank-control” features on the array. Data were background corrected and then normalized using quantile normalization [51]. Following normalization, a quality control step was performed that removed all data sets with a correlation coefficient less than 0.9 compared to the corresponding biological replicates. Of the original four biological replicate data sets representing cells grown in CDM, at least three were included in the analysis. The remaining probes of the biological replicates required intensity values greater than 300 on all three arrays. Regions that showed signals on probes of both strands were manually removed following the primary analysis. The statistical significance of the determined signals was tested by unpaired student’s t-test with a false discovery rate of 5%. Resulting data were combined with gene information from the flanking coding regions. Terminators and promoters were predicted by TransTermHP [52] ( http://transterm.cbcb.umd.edu/tt/Streptococcus_pyogenes_NZ131.tt) and BDGP Neural Network Promoter Prediction [53], and BProm ( http://www.SoftBerry.com), respectively. To investigate sRNA gene regulation, two biological replicates of growth experiments conducted in THY or BHI were included. Following data normalization, three-fold signal intensity differences between various conditions were determined using the GeneSpring GX (version 11) software (Agilent Technologies). A motif search was conducted using MEME Suite [27], followed by motif analyses using TOMTOM [28], ( http://meme.sdsc.edu/meme/intro.html).

### 5^′^ RACE

The transcriptional start sites of sRNA candidates were determined using 5^′^ RACE (Invitrogen) following the manufacturer’s instructions. Briefly, first strand cDNA was synthesized using gene-specific primers ( Additional file 2). The original mRNA was enzymatically removed and the 3^′^ end of the cDNA was tailed with dCTP by terminal deoxynucleotidyl transferase (TdT). PCR amplification was performed with nested, sequence-specific primers and an anchor primer provided by the 5^′^ RACE system. Primers specific for the sRNA genes tested here are listed in Additional file 2. Following amplification, PCR products were cloned into a TOPO-TA vector (Invitrogen) and sequenced (GATC Biotech AG).

### Quantitative reverse transcription PCR

Acidic phenol-extracted, DNaseI-treated total RNA was reverse transcribed to generate cDNA using the First-Strand cDNA Synthesis Kit from Invitrogen following the protocol provided by the manufacturer. For gene-specific reverse transcription (RT), three reactions were performed: two strand-specific reactions with either one forward or one reverse primer, and one control reaction without any primer. Primers were designed based on the full genome sequence of *S. pyogenes* M49 strain NZ131 (NCBI accession number: NC011375) and are listed in Additional file 3. Three independent RT experiments were performed and all subsequent PCR reactions were performed in triplicate. Primer efficiency was tested on genomic GAS M49 DNA prior to use in RT reactions. All cDNA products were amplified by PCR with two primers specific for the respective candidate sequences. Real time PCR amplification was performed with SYBR Green (Fermentas) using an ABI PRISM 7000 Sequence Detection system (Applied Biosystems). The level of 5S RNA gene transcription was used for normalization. Relative gene expression was determined by the ΔΔCT method [54].

### Northern blot analyses

Total RNA was isolated during exponential (OD_600_ = 0.4), transitional (OD_600_ = 0.8), and stationary (OD_600_ =1.2) growth phases. RNA samples (10 μg per growth phase) were loaded onto an 8% TBE-Urea polyacrylamide gel and separated by electrophoresis. Size standards (Ultra Low Range Ladder, Fermentas) were loaded on the same gel. RNA was electroblotted onto positively charged nylon membranes (Ambion), UV cross-linked, and probed overnight with a probe complementary to a candidate sRNA. Probes were generated by PCR with the same primers as used for the PCR reaction in qRT-PCR experiments (listed in Additional file 3). Probes were labelled with biotin prior to hybridization (Brightstar psoralen-biotin labeling kit, Ambion). A BrightStar BioDetect Kit (Ambion) was used for detection, and autoradiography films were exposed to the luminescent blots.

## Competing interests

We are currently applying for a patent relating to the small RNAs described in this manuscript. German Patent and Trade Mark Office (DPMA), official file number: 10 2012 104 814.2.

## Authors’ contributions

NP participated in the design of the study, carried out experiments, analysed the microarray data, and drafted the manuscript. JN and AWK carried out experiments. VB carried out the array probe hybridisation and participated in writing the manuscript. PR performed data analyses using the MOSES bioinformatics tool. AB and TH^2^ participated in the design of the study and helped with data analysis and interpretation. JR and TH^4^ helped with data integration, analysis and interpretation. BK conceived of the study, participated in its design and coordination, and participated in writing the manuscript. All authors read and approved the final manuscript.

## Supplementary Material

Additional file 1 A: northern blot analysis of CRISPR gene expression and transcript processing in GAS M49; B: northern blot analysis of sRNASpy490957c gene expression in GAS M49. Click here for file

Additional file 2 Sequences of primers used for 5^′^ RACE. Click here for file

Additional file 3 Sequences of gene-specific primers employed for qRT-PCR. Click here for file
