# Supplementary figures and images for: Identification of novel growth phase- and media-dependent small non-coding RNAs in Streptococcus pyogenes M49 using intergenic tiling arrays
Source: BMC Genomics. 2012 Oct 13;13:550. doi: 10.1186/1471-2164-13-550 (PMC3542284; doi:10.1186/1471-2164-13-550)

**A**

sRNASpy490822/tracrRNA

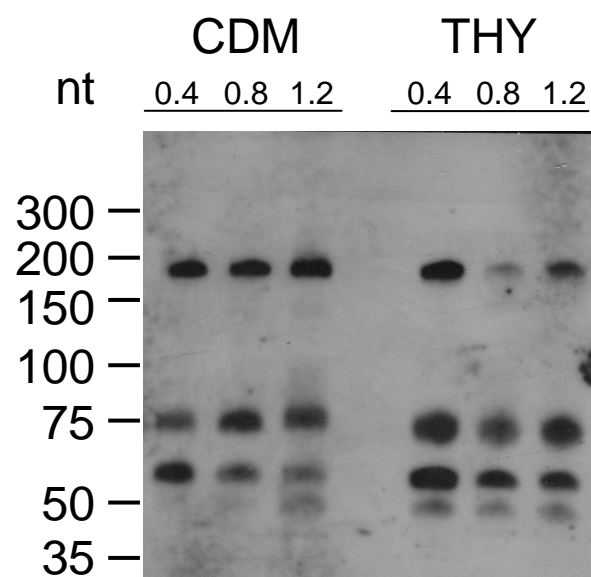

**B**

sRNASpy490957c

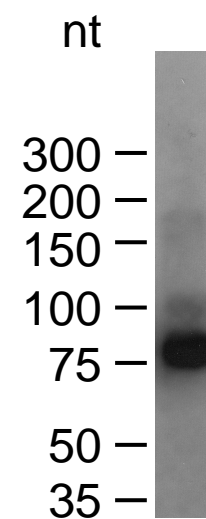

Supplement: Additional file 1 — A: northern blot analysis of CRISPR gene expression and transcript processing in GAS M49; B: northern blot analysis of sRNASpy490957c gene expression in GAS M49. [file 1471-2164-13-550-S1.pdf]
